# Assessment of Moisture Influence on the Thermal Properties of Autoclaved Aerated Concrete of Four Density Classes

**DOI:** 10.3390/ma19214724

**Published:** 2026-11-06

**Authors:** Halina Garbalińska, Magdalena Bochenek

**Affiliations:** Faculty of Civil and Environmental Engineering, West Pomeranian University of Technology in Szczecin, al. Piastów 17, 70-310 Szczecin, Poland; magdalena.bochenek@zut.edu.pl

**Keywords:** autoclaved aerated concrete, moisture, thermal properties, thermal conductivity, volumetric heat capacity

## Abstract

Autoclaved aerated concrete (AAC) is currently one of the most popular building materials used for the construction of external walls in modern buildings. Current global market forecasts indicate that the value of the AAC sector will increase from USD 28.91 billion in 2026 to USD 50.04 billion by 2034. This trend coincides with the progressively more stringent thermal performance requirements imposed on external building envelopes. In response to these trends, a comprehensive investigation was undertaken to determine the basic thermal parameters of the most commonly used aerated concretes with densities ranging from 400 to 700 kg/m^3^. The values of these parameters primarily depend on the pore structure of these materials, but even more so on their moisture content. For the studies, AAC400, AAC500, AAC600, and AAC700 aerated concretes from the same manufacturer and with the same raw-material composition were used. For each of the four selected concretes, initial microstructural tests were carried out to obtain detailed data on pore distribution in each of the tested materials. In the main experiment, first, prismatic specimens of all four classes were subjected to controlled capillary absorption, which led to varying moisture distribution. After one month of capillary uptake, these specimens were cut along their height into 6 component samples. Then, on these component samples, with different moisture levels, thermal parameters were measured: the thermal conductivity coefficient *λ* and the volumetric heat capacity *c*_v_. The measurements were done using a non-stationary technique with the ISOMET 2104 apparatus. Immediately after testing, the samples were dried to determine the moisture level of each sample: their mass moisture content (*U*_m_) and volumetric moisture content (*U*_v_). The results of the conducted studies demonstrate that even a moderate increase in moisture content leads to a substantial rise in both thermal conductivity *λ* and volumetric heat capacity *c*_v_ for all tested materials (AAC400, AAC500, AAC600, AAC700). This confirms the necessity of incorporating moisture effects into calculations related to both the thermal insulation performance and the thermal dynamics of AAC-based building envelopes. The study shows that these relationships can be effectively described using the proposed linear functions, which provide high accuracy for *λ*(*U*_v_) and *λ*(*U*_m_) with R^2^ ≥ 0.99, as well as for *c*_v_(*U*_v_) and *c*_v_(*U*_m_) with R^2^ ≥ 0.92.

## 1. Introduction

In recent years, legal regulations introduced within the European Union have obligated Member States to construct buildings with nearly zero energy consumption. Standardized requirements have also been established for thermal and hygrothermal performance, which must be considered at the building design stage.

The scale of changes that have taken place over the past decades is most clearly reflected in the strict lowering of the allowable values for the heat transfer coefficients of building envelopes. The value of this coefficient is mainly influenced by the thickness of the individual layers of the partition and by the thermal conductivity values of the materials from which these layers are made.

Because of this, building material manufacturers, in order to meet growing requirements, are also making efforts to lower the thermal conductivity values of their materials. Article [1] illustrates how the thermal conductivity coefficient of different autoclaved aerated concretes changed over several decades.

Autoclaved aerated concrete (AAC) is a material that has good strength properties but at the same time has relatively high thermal insulation. AAC has been the subject of extensive research, as it is the most widely used wall-building material in both Poland and Europe. Its importance is also increasing in the United States, where timber remains the predominant wall material. However, in regions exposed to hurricanes and other climate-related disasters, there is a growing demand for more resilient construction materials, which contributes to the rising popularity of AAC.

According to [2], the global autoclaved aerated concrete market size was valued at USD 26.99 billion in 2025. Forecasts indicate that this market will grow from USD 28.91 billion in 2026 to USD 50.04 billion by 2034. Market analyses show increasing interest in AAC among contractors, architects, and developers who seek cost-effective solutions offering enhanced durability and favorable thermal insulation properties.

**Figure 1:**
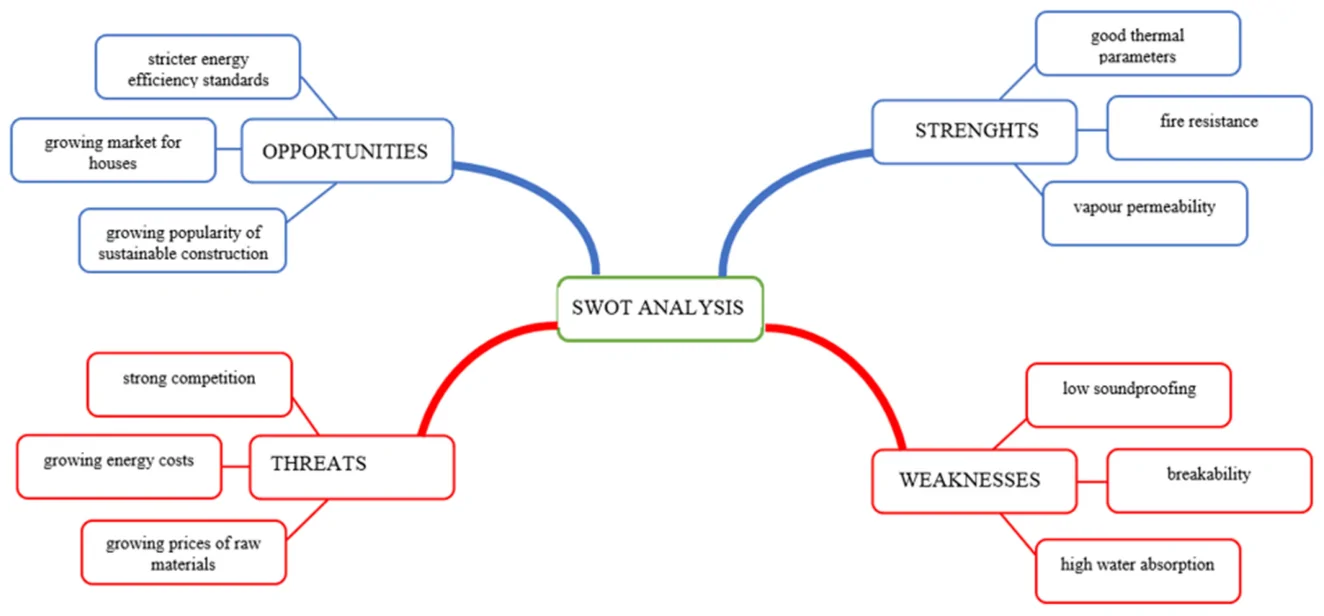
Autoclaved aerated concrete in the construction market—SWOT analysis, own study.

The conducted SWOT analysis of autoclaved aerated concrete (AAC) in the construction market indicates its strong position, driven by its thermal insulation performance, ease of processing, and energy efficiency, while also highlighting challenges related to moisture absorption and competition from alternative technologies. Figure 1 presents the SWOT analysis of AAC, prepared using a mind-map technique.

The thermal properties of building materials are strongly dependent on their moisture content. Therefore, it is important not only to know the thermal parameters declared by manufacturers but also how they change with moisture levels. This issue is especially relevant for autoclaved aerated concrete, due to its high porosity and water absorption. Such relationships can be determined through a series of laboratory tests. However, these tests are very labor-intensive and time-consuming. Nevertheless, because of the popularity of autoclaved aerated concrete and the importance of this problem, numerous researchers have undertaken such investigations and subsequently proposed descriptions of the moisture-dependent behavior of thermal parameters, primarily thermal conductivity.

The literature provides various approaches for describing the relationship between thermal conductivity and moisture content. For example, in [3], the dependence of thermal conductivity on moisture content is expressed by the following equation:

*λ*_(w)_ = *λ*_ma*t*_(1 − *P*) + *λ*_wat_*w* + *λ*_a_(*P* − *w*). (1)

Thermal conductivity coefficients can be described separately within the hygroscopic and over-hygroscopic moisture ranges by applying appropriate formulas, or alternatively by using nonlinear equations.

In [3], the above relationship was divided into two independent expressions. As a result, the thermal conductivity coefficient was defined as follows:

- In the area of hydroscopic moisture:

*λ*_(w)_ = *λ*_mat(u,ε)_(1 − *P*) + *λ*_wat_(*w* − *w*_min_) + *λ_a_*(*P* − *w*), (2)

- Outside the area of hydroscopic moisture:

(3) $$\lambda_{\left(w\right)}=\lambda_{\mathrm{mat}(u)}\left(1-P\right)+\lambda_{\mathrm{wat}}\;\sum_{f}w_{f}R_{f}+\lambda_{a}(P-w).$$

In the formulas, *λ*_(w)_—thermal conductivity of the wet material [W/(m·K)]; *λ*_mat_—thermal conductivity of the material matrix without pores [W/(m·K)]; *λ*_a_—thermal conductivity of air [W/(m·K)]; *λ*_wat*—*_thermal conductivity of water [W/(m·K)]; *P*—volume porosity [–]; *w—*material moisture content [–]; *w*_sat—_moisture content at saturation [–]; *f*—number of considered pore fractions; and *w*_f_ *R*_f—_autocorrelation function.

In WTA [4], the following relationship was proposed:

(4) $$\lambda_{\left(w\right)}=\lambda_{o}\;\cdot \;(1+\frac{b}{100}\cdot \frac{w}{\rho_{s}}\cdot 100),$$

where *λ*_(w)_—thermal conductivity of the wet material [W/(m·K)]; *λ*_0_—thermal conductivity of the dry material [W/(m·K)]; *ρ*_s_—material density in dry state [kg/m^3^]; *w*—total water content [kg/m^3^]; and *b*—thermal conductivity increment.

The increment *b* indicates the percentage increase in thermal conductivity corresponding to a percentage increase in mass moisture content.

Due to the importance of the problem, standard formulas are currently available that describe the relationship between the thermal conductivity coefficient and moisture content. In standard [5], a formula is given in the following form:

*λ*_2_ = *λ*_1_∙*F*_m_, (5)

where *F*_m_—moisture conversion factor [–].

The conversion factor *F_m_* can be determined in two ways:

(a) Conversion of moisture content expressed as a mass-to-mass ratio:

(6) $$F_{m}=e^{f_{u}(u_{2}-u_{1})},$$

where

*f*_u_—moisture-conversion coefficient expressed as a mass-to-mass ratio;

*u*_1_—moisture content expressed as a mass-to-mass ratio for the first set of conditions;

*u*_2_—moisture content expressed as a mass-to-mass ratio for the second set of conditions.

(b) Conversion of moisture content expressed as a volume-to-volume ratio:

(7) $$F_{m}=e^{f_{\Psi }(\Psi_{2}-\Psi_{1})},$$

where

*f*_Ψ_—moisture-conversion coefficient expressed as a volume-to-volume ratio;

*Ψ*_1_—moisture content expressed as a volume-to-volume ratio for the first set of conditions;

*Ψ*_2_—moisture content expressed as a volume-to-volume ratio for the second set of conditions.

Article [6] presents the theoretically determined courses of thermal conductivity variation with moisture content. The calculations were performed using EN ISO 10456 [5], and the resulting plots cover the range of mass moisture content *U*_m_ from 4% to 45%. They refer to six autoclaved aerated concretes differing in density, ranging from 350 kg/m^3^ to 700 kg/m^3^.

Simultaneously, in [6], a comparison was performed and significant differences were demonstrated between the results obtained from experimental studies and the results of standard calculations.

Study [7] presented laboratory tests of the thermal conductivity coefficient of aerated concrete blocks of class 350 and 600 kg/m^3^ in a post-production wet state with moisture content *U*_m_ = 40% and *U*_m_ = 45%, as well as in a dry state. In the experiment, the stationary method was used. Based on the conducted tests, it was also demonstrated that the standardized conversion coefficient *f*_m_ = 4 [kg/kg] is significantly overestimated, and that the relationship between thermal conductivity and the material’s mass moisture content is linear rather than exponential, as specified in standard [5].

In [8], autoclaved aerated concretes of classes 300–700 kg/m^3^ were tested, and it was found that for AAC materials with densities between 400 and 700 kg/m^3^, the increase in thermal conductivity caused by moisture content can be described using a linear relationship. The coefficient of determination R^2^ indicated a satisfactory quality of fit between the adopted model and the experimental data. Only for AAC with a density of 300 kg/m^3^ did the coefficient of determination reach a value of 0.86, meaning that the linear description was less precise.

Extensive research on autoclaved aerated concrete has been conducted at Czech Technical University. For example, in [9], results were presented for AAC materials with densities of 300, 350, and 500 kg/m^3^. Thermal parameters were measured using a transient method. The authors of [9] proposed different functional forms to describe the dependence of thermal conductivity on moisture content. For AAC classes 300 and 500, an exponential function was applied (R^2^ = 0.9404 and R^2^ = 0.9174, respectively), whereas for AAC class 350, a linear function was used (R^2^ = 0.972).

The results obtained in [9] were referenced, among others, in [10], where attention was drawn to the necessity of recalculating thermal parameters with respect to the material’s moisture content.

Paper [11] discussed guidelines and standards from different countries that take material moisture into account in the design process. It was concluded that the currently applicable guidelines are insufficient.

In paper [12], the standard relationships were described, similar to [11]. The difference is that paper [12] discussed the standards used in Poland. It was found that incorrectly stating the thermal conductivity values in the declarations of performance is not uncommon. The resulting errors in assessing the actual thermal insulation of building partitions may reach up to 25%.

Nowadays, thanks to the availability of specialized software, calculations are carried out not only for thermal insulation but also for the thermal dynamics of building partitions. In the first case, thermal conductivity is the key factor, while in the second case, the heat capacity of the materials used and their moisture state.

It should be emphasized that numerous studies have investigated the variation in thermal conductivity of autoclaved aerated concrete with moisture content. However, only sporadically have researchers attempted to identify similar relationships for thermal capacity.

Results of such investigations were presented in [13]. The authors tested two AAC classes, 400 and 500 kg/m^3^. The measurements covered both thermal conductivity and heat capacity, and were conducted at different moisture contents in the tested samples. This allowed the reconstruction of the dependence of both parameters on moisture level. In each case, a linear function was proposed to describe the relationship.

In [14], simultaneous investigations were carried out to determine the dependence of both thermal conductivity and heat capacity on moisture content. In each case, a linear function was proposed to describe these relationships, although the linear fit for heat-capacity dependence on moisture exhibited rather weak agreement, with R^2^ = 0.53.

Another study that includes a description of research on the effect of moisture in the material on both its thermal conductivity and heat capacity is work [15]. Experimental tests were performed on autoclaved aerated concrete produced using fly-ash technology, with densities of 420, 480, 520, 560, and 610 kg/m^3^. To examine the effect of moisture content on compressive strength and thermal properties, water was added to the samples prior to measurements in quantities ensuring mass moisture contents of 1, 3, 5, 10, 20, and 30%. The samples were stored in tightly sealed plastic bags for two weeks. Thermal parameters were tested using the non-stationary method with an ISOMET 2104 instrument. The results described in work [15] confirmed the significant impact of moisture content on the physical and mechanical properties of autoclaved aerated concrete.

In our own studies presented below, we undertook an assessment of the impact of varying moisture levels on the two basic thermal parameters of the most commonly used aerated concretes, with densities ranging from 400 to 700 kg/m^3^. For each of the four selected concretes, measurements of thermal conductivity and volumetric heat capacity were carried out on samples with different moisture levels. The aim was to determine, for the selected aerated concretes, whether a linear function is sufficient in every case to accurately describe the relationship, or if it is necessary to use an exponential function or another model to ensure correctness in describing both the dependence of thermal conductivity on moisture and the dependence of volumetric heat capacity on moisture.

## 2. Materials and Methods

### 2.1. Material Used in the Research

The investigations were carried out on autoclaved aerated concrete samples of four density classes: 400, 500, 600, and 700 kg/m^3^. Table 1 summarizes their basic properties, taken from the manufacturer’s declaration.

All concretes were sourced from the same manufacturer and exhibited identical raw-material composition, which was confirmed through independent testing. The mineral composition was determined using X-ray diffraction. The percentage phase contributions obtained for all tested classes are presented in [16].

Next, as part of work [17], a series of microstructural studies were carried out to determine the differences in porosity structure of the different classes.

**Figure 2:**
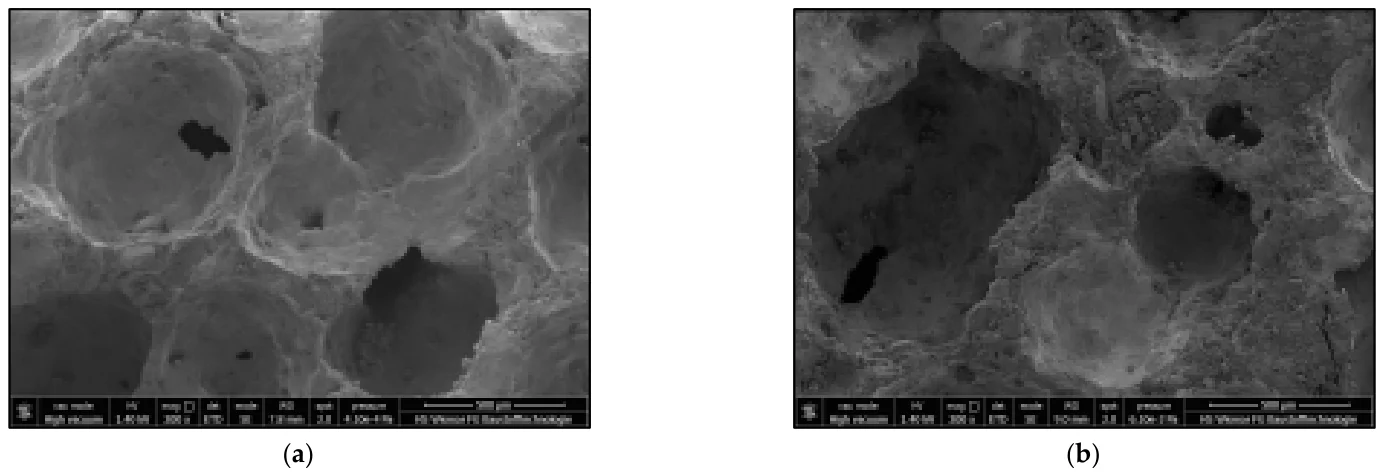
Microscopic images of the pore structures at 200 times magnification: (**a**) AAC 500, (**b**) AAC 700.

**Figure 3:**
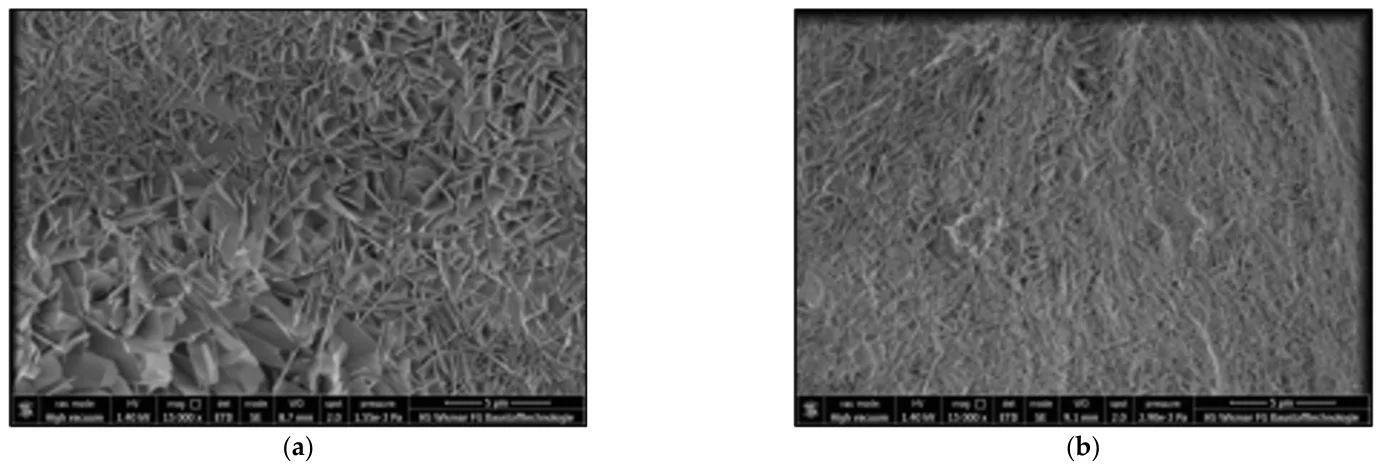
Microscopic images of the pore structures at 15,000 times magnification: (**a**) AAC 500, (**b**) AAC 700.

Figure 2 and Figure 3 show example images obtained using SEM, illustrating the structure of AAC 500 and AAC 700 at 200× and 15,000 magnifications.

To obtain more detailed information on the pore-structure characteristics of the tested concretes, porosimetric measurements were performed. The investigations were carried out using a PoreMaster porosimeter (Quantachrome Instruments/Anton Paar QuantaTec), Boynton Beach, FL, USA, which enables the determination of the volume and surface area of macropores and mesopores, as well as the assessment of their size distribution.

**Figure 4:**
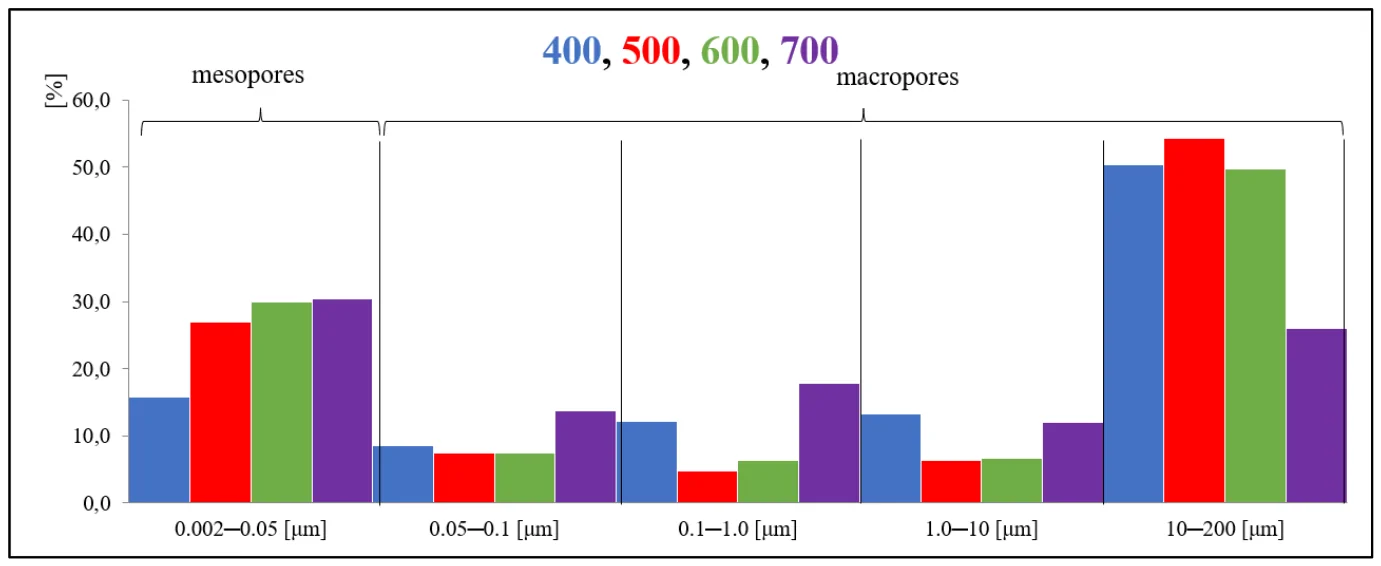
The percentage distribution of pores in different ranges in autoclaved aerated concretes of classes 400, 500, 600, and 700.

Based on the obtained results, the percentage contribution of individual pore-size ranges in the analyzed autoclaved aerated concretes was determined. These data are illustrated graphically in Figure 4.

In paper [18], differential curves of pore size distribution were presented for all four aerated concretes tested in [17].

Independently of the thermal-parameter measurements reported in the present article, earlier publications have provided results obtained for the same materials concerning the dependence of their diffusivity on moisture content, e.g., [18], as well as the determination of desorption isotherms using both the traditional method and the DVS technique [19], sorption isotherms [20], and the application of various models to describe the sorption isotherms of this group of autoclaved aerated concretes (AAC400, AAC500, AAC600, and AAC700) [21].

### 2.2. Research Methodology

In practical conditions, aerated concrete in exterior walls usually shows higher moisture content compared to the conditions assumed in tests conducted by the manufacturer. As a result, the actual thermal properties of this material differ from the values declared by the manufacturer. In many cases, the magnitude of these changes is substantial, manifesting as a significant increase in both the real thermal-conductivity coefficient and the heat capacity.

For this reason, an experiment was undertaken to determine how increasing moisture content translates into increases in both parameters for the most commonly used AAC density classes. The investigations were performed on autoclaved aerated concrete samples of classes 400, 500, 600, and 700 kg/m^3^ at various moisture levels.

Initially, prismatic specimens of 12 cm × 12 cm × 24 cm were prepared and sealed on their lateral surfaces with transparent silicone to ensure unidirectional water transport and to prevent uncontrolled drying through the side faces. The frontal surfaces were left unsealed—allowing the lower surface to remain in continuous contact with water, while the upper surface could release air gradually displaced from the pore network by the incoming water. The samples, sealed on their lateral faces, were placed in trays filled with distilled water. Spacers were positioned at the bottom of the trays to provide point support and ensure uniform positioning of the specimens relative to the tray bottom. The samples were immersed to a depth of approximately 2–5 mm, and a constant immersion level was maintained throughout the entire testing period.

**Figure 5:**
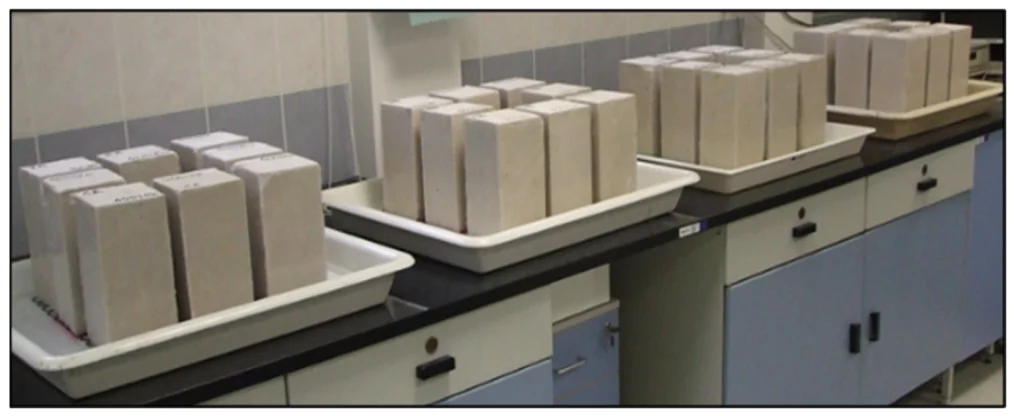
View of prismatic specimens (12 cm × 12 cm × 24 cm) of AAC 400, AAC 500, AAC 600, and AAC 700 during the process of capillary absorption.

The investigations were conducted in an air-conditioned laboratory at a constant temperature of approximately 20 °C and relative humidity of about 40%. A view of the first stage of the experiment is presented in Figure 5.

The investigations into the dependence of thermal parameters on moisture content were initiated after a one-month period of capillary uptake, which resulted in a variable moisture distribution along the height of the prismatic specimens placed in the trays.

For each type of autoclaved aerated concrete, three prismatic specimens were selected. Each specimen was then cut along its height into six samples of equal thickness, approximately 4 cm. Immediately after cutting, the 12 cm × 12 cm × 4 cm samples were wrapped in aluminum foil to prevent moisture loss and stored in airtight containers. Each of these subsamples exhibited a different moisture level.

In subsequent stages of the experiment, measurements of thermal parameters, namely thermal conductivity and volumetric heat capacity, were performed on such cut samples. Thermal properties tests were carried out using a non-stationary method. The ISOMET 2104 measuring device was used, which is designed for direct measurement of the thermophysical properties of a wide range of isotropic materials. The device ISOMET 2104 thermal properties analyzer (Applied Precision Ltd.), Bratislava, Slovakia is equipped with two optional types of measurement probes: needle probes for soft materials and surface probes for hard materials.

Two surface probes with the following measurement ranges were used in the conducted studies:

Surface Probe API 210411: *λ* = 0.04–0.30 [W/(m·K)], *c*_v_ = 4.0 × 10^4^–1.5 × 10^6^ [J/(m^3^·K)];

Surface Probe API 210412: *λ* = 0.30–2.00 [W/(m·K)], *c*_v_ = 1.5 × 10^6^–4.0 × 10^6^ [J/(m^3^·K)].

The measurement is based on the analysis of the temperature response of the tested material to heat flow impulses. Heat flow is emitted by an electric resistor located in the probe, which is applied to the sample being tested. Measurement data are stored in the high-capacity internal memory. The provided software package enables updating of calibration coefficients after recalibration of measurement probes using reference materials. More detailed data can be found on the manufacturer’s website (Applied Precision). A method was chosen where the measurement time is around 15 min. This allowed avoiding changes in the material’s moisture content that occur during long-term measurements with stationary methods.

**Figure 6:**
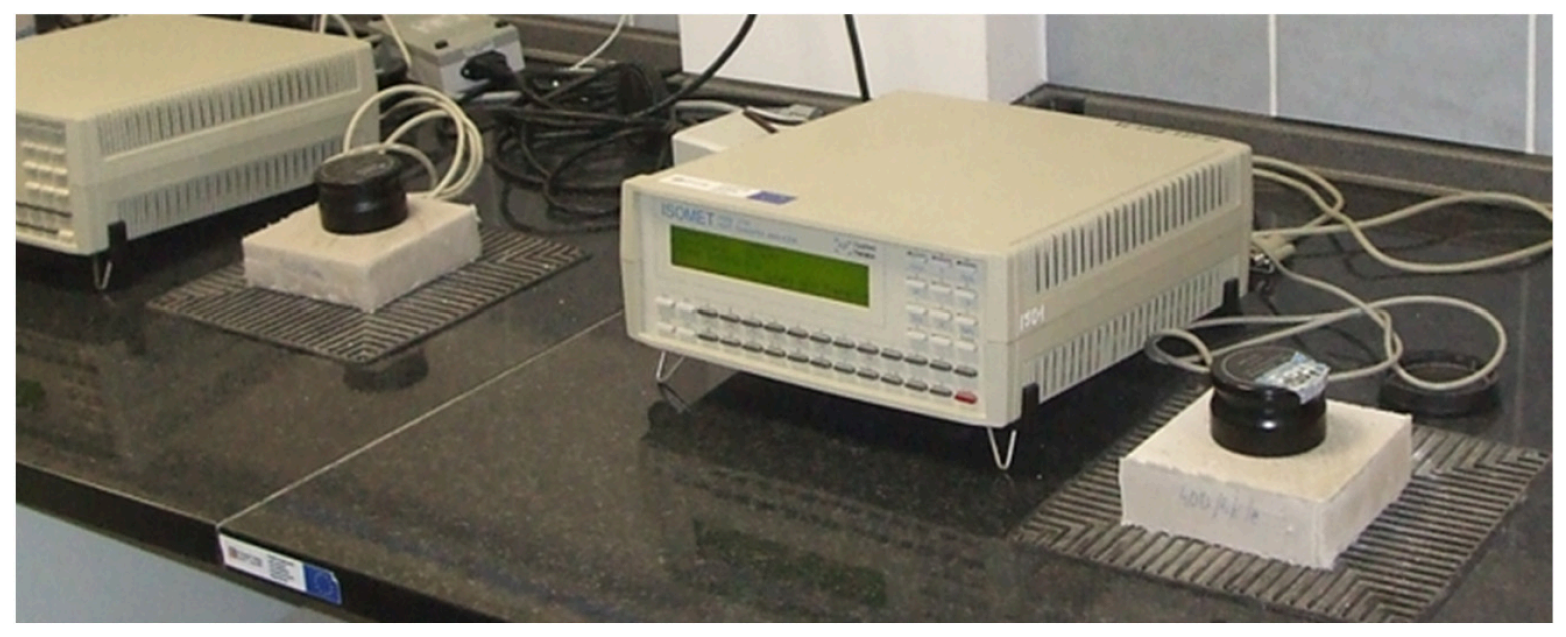
View of the measuring equipment (ISOMET 2104) during thermal-parameter measurements.

Each measurement was carried out twice, and the results were recorded automatically by the measuring instrument, shown in Figure 6.

Immediately before the thermal measurements, each sample was weighed and its bulk density at the current moisture level was determined. After completing the thermal-property measurements, the samples were placed in an oven (POL-EKO® A. Polok-Kowalska sp.k., Wodzisław Śląski, Poland) at 105 °C and dried to constant mass. Their mass and dry bulk density were then determined again. For each sample, both volumetric and mass moisture contents were established.

## 3. Results

**Figure 7:**
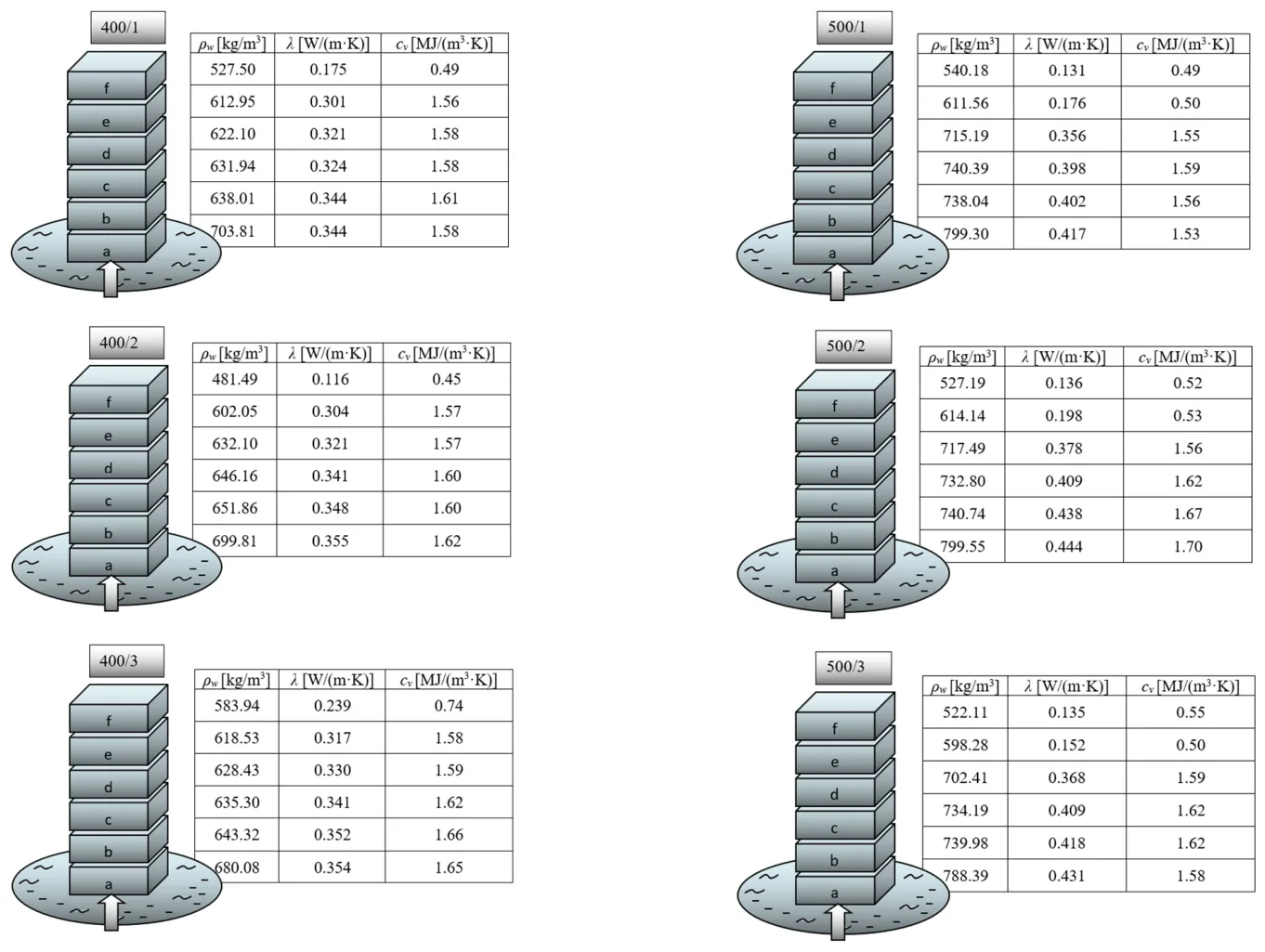
Schematic diagram of 12 cm × 12 cm × 24 cm blocks division for samples 12 cm × 12 cm × 4 cm, AAC 400 and AAC 500.

**Figure 8:**
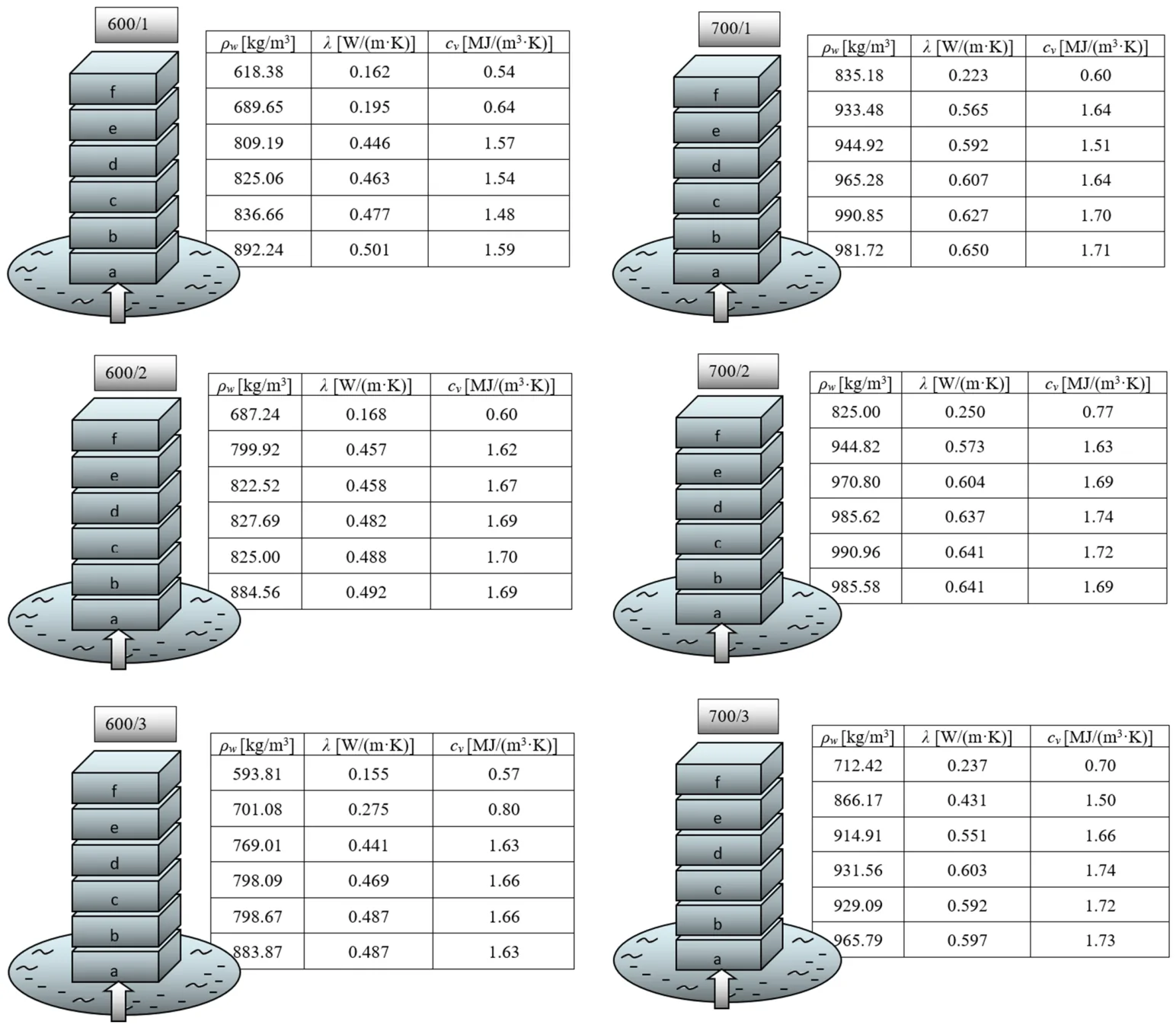
Schematic diagram of 12 cm × 12 cm × 24 cm blocks division for samples 12 cm × 12 cm × 4 cm, AAC 600 and AAC 700.

Figure 7 and Figure 8 show the division of 12 cm × 12 cm × 24 cm block samples into 12 cm × 12 cm × 4 cm component samples. Additionally, the average values of density, thermal conductivity, and volumetric heat capacity for each component sample are provided.

The experiment provided extensive data enabling the determination of functional relationships between volumetric moisture content *U*ᵥ and mass moisture content *U*_m_, and the thermal conductivity coefficient *λ* and volumetric heat capacity *c*_v_.

**Figure 9:**
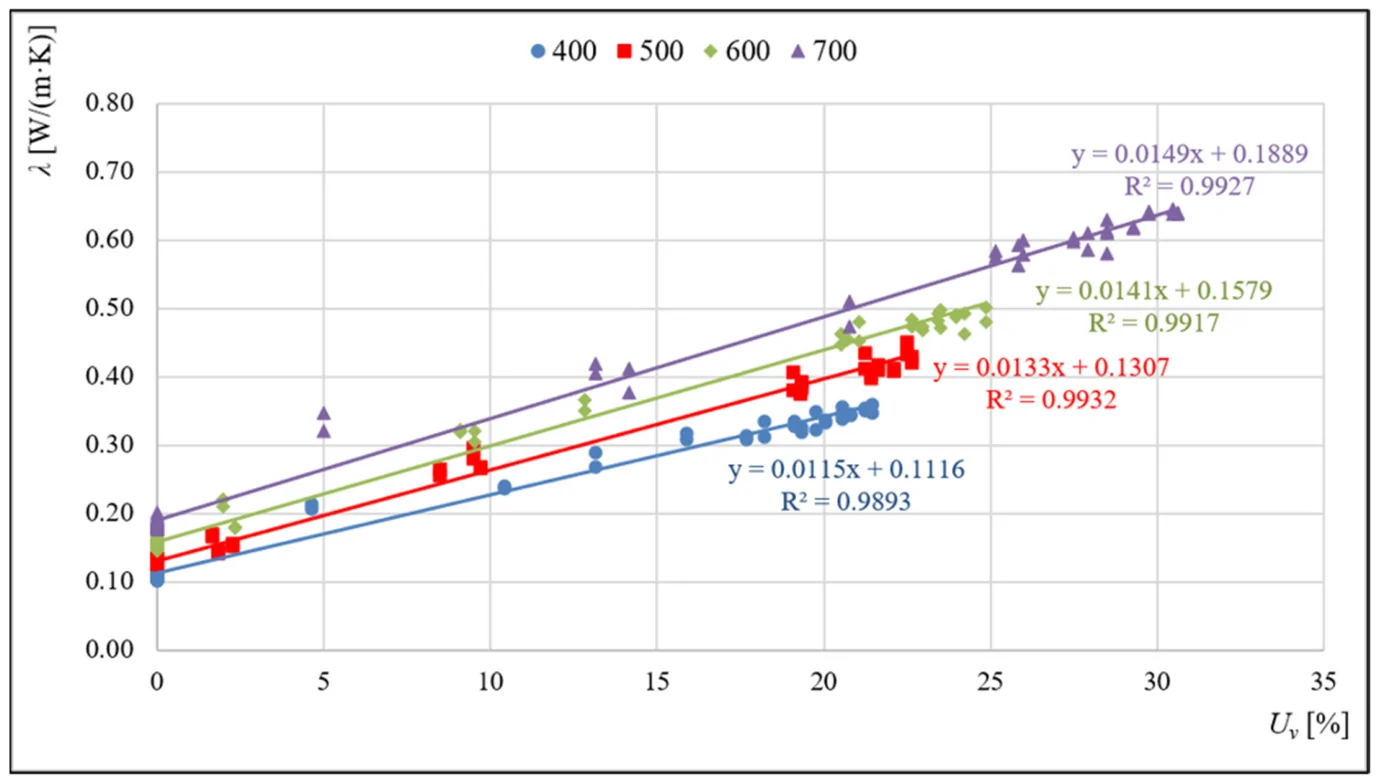
Thermal conductivity *λ* of autoclaved aerated concrete of classes 400, 500, 600, and 700 kg/m^3^ as a function of moisture content *U*_v_.

**Figure 10:**
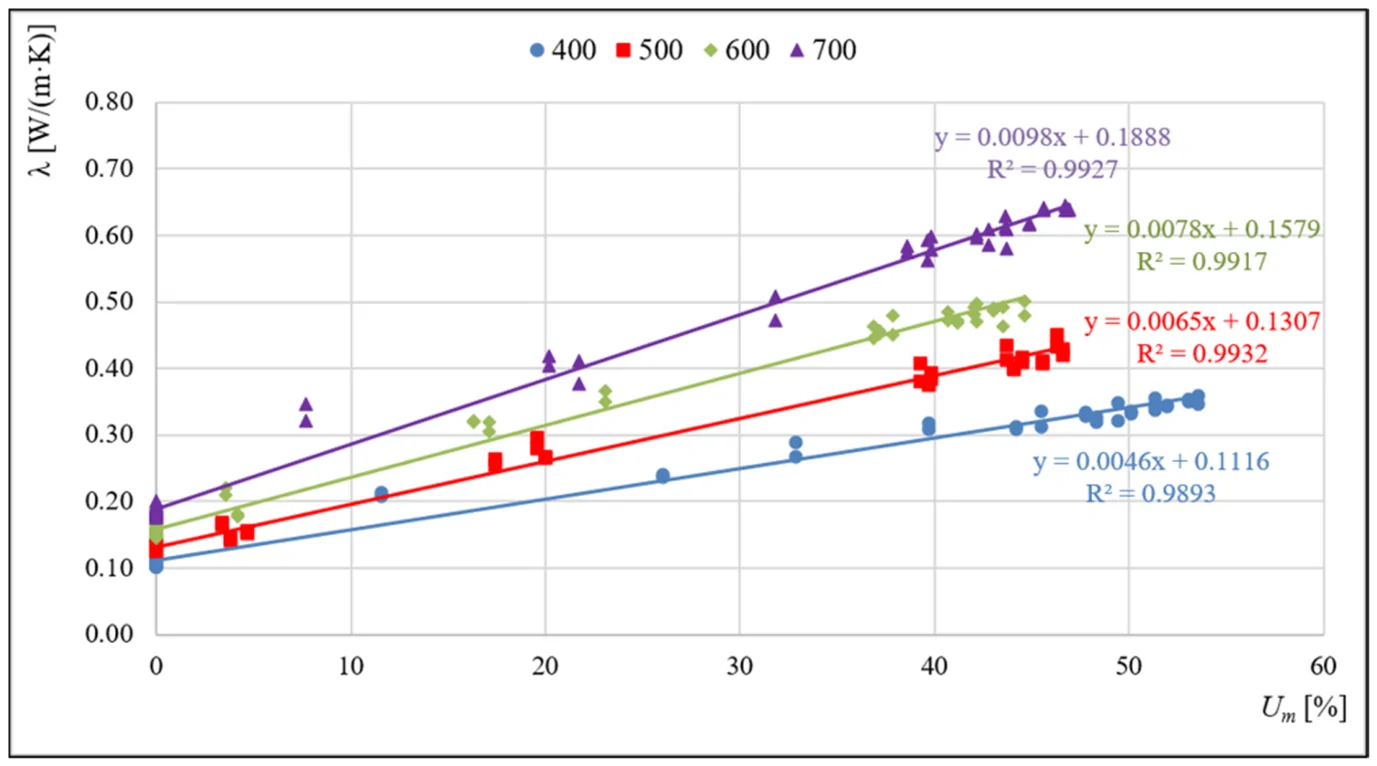
Thermal conductivity *λ* of autoclaved aerated concrete of classes 400, 500, 600, and 700 kg/m^3^ as a function of moisture content *U*_m_.

Figure 9 and Figure 10 present the plots of *λ* = *f(U*ᵥ*)* and *λ* = *f(U*_m_*)*, respectively, for all four tested density classes: 400, 500, 600, and 700 kg/m^3^.

Based on the obtained results, the dependence of the thermal conductivity coefficient *λ* on volumetric moisture content *U*ᵥ can be expressed by the following equation:

*λ*(*U*_v_) = *A*·*U*_v_ + *λ*_0_, (8)

where *λ*_0_—thermal conductivity of the dry material [W/(m·K)]; *U*ᵥ*—*volumetric moisture content [%]; and *A*—slope coefficient.

The dependence of the thermal conductivity *λ* on mass moisture content *U*_m_ can be expressed by the following equation:

*λ*(*U*_m_) = *B*·*U*_m_ + *λ*_0_, (9)

where *λ*_0_—thermal conductivity of the dry material [W/(m·K)]; *U*_m_—mass moisture content [%]; and *B*—slope coefficient.

The value of the intercept in Equations (8) and (9) defines the thermal conductivity of autoclaved aerated concrete in a completely dry state and is equal to (for both volumetric and mass moisture content)

- AAC 400: *λ*_0_ = 0.1116 [W(m∙K)];
- AAC 500: *λ*_0_ = 0.1307 [W(m∙K)];
- AAC 600: *λ*_0_ = 0.1579 [W(m∙K)];
- AAC 700: *λ*_0_ = 0.1888 [W(m∙K)].

All regression equations exhibit a very high coefficient of determination R^2^:

- AAC 400: R^2^ = 0.9893;
- AAC 500: R^2^ = 0.9932;
- AAC 600: R^2^ = 0.9917;
- AAC 700: R^2^ = 0.9927.

The slope of the regression lines (their directional coefficient) indicates how the thermal conductivity of autoclaved aerated concrete increases with each 1% rise in moisture content.

For volumetric moisture content *U*ᵥ, the slope coefficient for the respective density classes is

- AAC 400: *A* = 0.0115;
- AAC 500: *A* = 0.0133;
- AAC 600: *A* = 0.0141;
- AAC 700: *A* = 0.0149.

For mass moisture content *U*_m_, the slope coefficient for the respective density classes is

- AAC 400: *B* = 0.0046;
- AAC 500: *B* = 0.0065;
- AAC 600: *B* = 0.0078;
- AAC 700: *B* = 0.0098.

**Figure 11:**
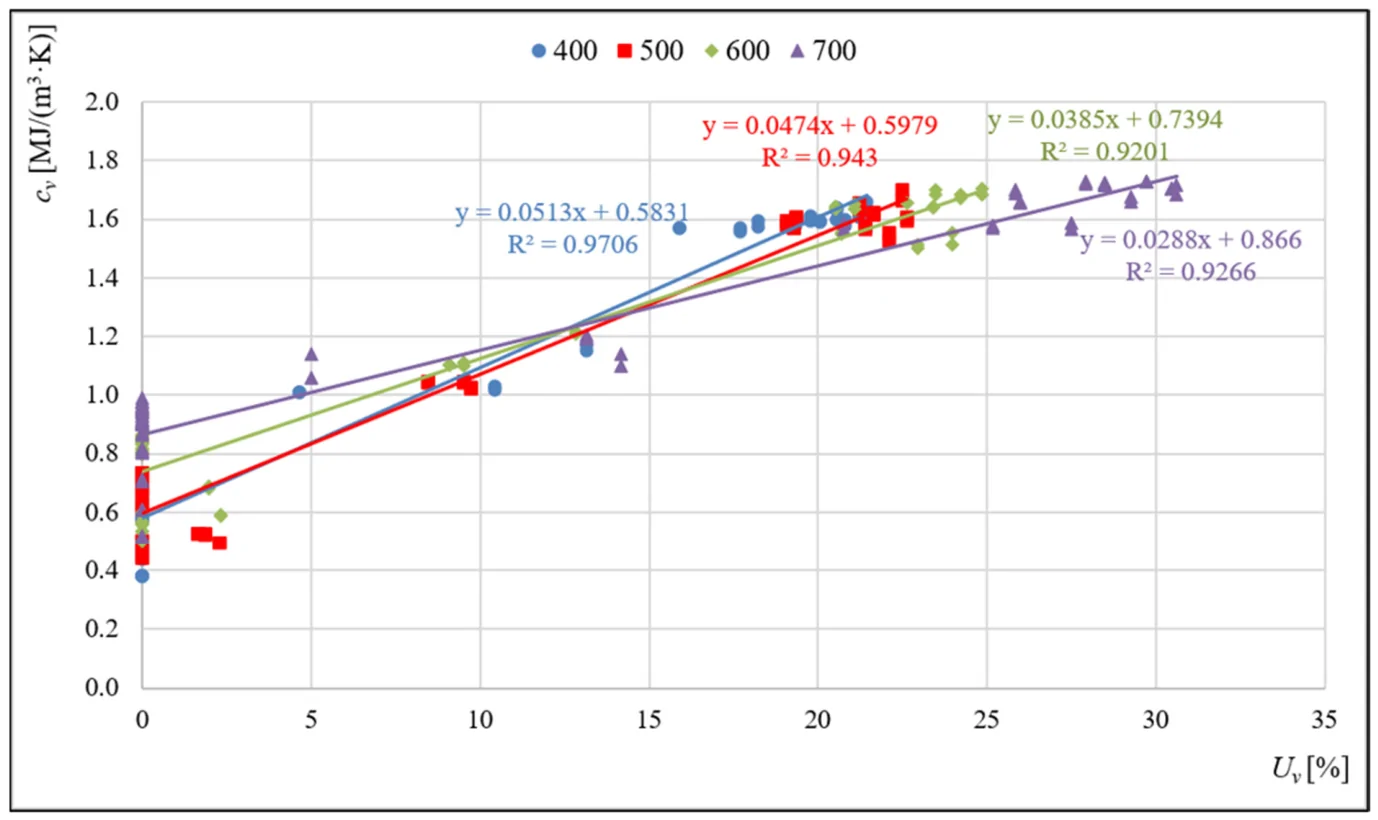
Volumetric heat capacity *c*_v_ of autoclaved aerated concrete of classes 400, 500, 600, and 700 kg/m^3^ as a function of moisture content *U*_v_.

**Figure 12:**
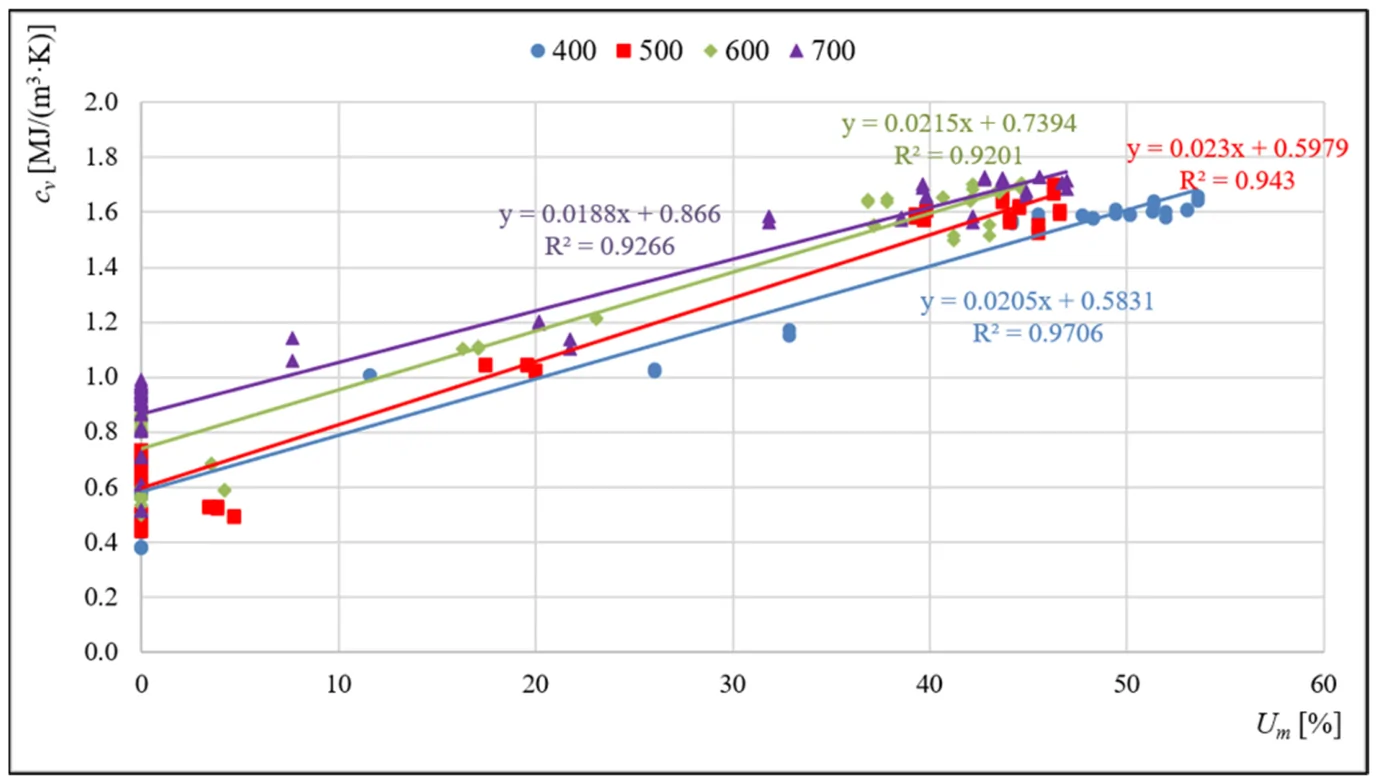
Volumetric heat capacity *c*_v_ of autoclaved aerated concrete of classes 400, 500, 600, and 700 kg/m^3^ as a function of moisture content *U*_m_.

Figure 11 and Figure 12 present the plots of *c*_v_ = *f*(*U*_v_) and *c*_v_ = *f*(*U*_m_), respectively, for all four tested density classes: 400, 500, 600, and 700 kg/m^3^.

The obtained results allow expressing the relationship of the volumetric heat capacity *c*ᵥ to the volumetric moisture content *U*ᵥ using the following equation:

*c*_v_(*U*_v_) = *C*·*U*_v_ + *c*_vo_, (10)

where *c*ᵥ_o_—volumetric heat capacity of the dry material [J/(m^3^·K)]; *U*ᵥ—volumetric moisture content [%]; and *C*—slope coefficient.

The dependence of volumetric heat capacity *cᵥ* on the mass moisture content *U*_m_ of the material can be expressed by the following equation:

*c*_v_(*U*_m_) = *D*·*U*_m_ + *c*_vo_, (11)

where *c*ᵥ_o_—volumetric heat capacity of the dry material [J/(m^3^·K)]; *U*_m_—mass moisture content [%]; and *D*—slope coefficient.

The value of the intercept in Equations (10) and (11) defines the volumetric heat capacity of autoclaved aerated concrete in a completely dry state and is equal to (for both volumetric and mass moisture content)

- AAC 400: *c*_vo_ = 0.5831 [MJ(m^3^∙K)];
- AAC 500: *c*_vo_ = 0.5979 [MJ(m^3^∙K)];
- AAC 600: *c*_vo_ = 0.7394 [MJ(m^3^∙K)];
- AAC 700: *c*_vo_ = 0.8660 [MJ(m^3^∙K)].

All regression equations exhibit a very high coefficient of determination R^2^:

- AAC 400: R^2^ = 0.9706;
- AAC 500: R^2^ = 0.9430;
- AAC 600: R^2^ = 0.9201;
- AAC 700: R^2^ = 0.9266.

The slope of the regression lines (their directional coefficient) indicates how the volumetric heat capacity of autoclaved aerated concrete increases with each 1% rise in moisture content.

For volumetric moisture content *U*ᵥ, the slope coefficient for the respective density classes is

- AAC 400: *C* = 0.0513;
- AAC 500: *C* = 0.0474;
- AAC 600: *C* = 0.0385;
- AAC 700: *C* = 0.0288.

For mass moisture content *U*_m_, the slope coefficient for the respective density classes is

- AAC 400: *D* = 0.0205;
- AAC 500: *D* = 0.0230;
- AAC 600: *D* = 0.0215;
- AAC 700: *D* = 0.0188.

## 4. Conclusions

The four tested classes of autoclaved aerated concrete (AAC400–AAC700) differed significantly in density and, consequently, in pore structure. Regardless of this variation, all four classes confirmed that the changes in their basic thermal parameters due to moisture could be described using linear functions.

It was found that, for all the tested AAC classes, the thermal conductivity coefficient *λ* increases linearly with rising volumetric and mass moisture content. The relationship is clearly linear, with a very good agreement (R^2^ > 0.989).

The thermal conductivity values determined in the dry state differed considerably among the classes, ranging from *λ*_0_ = 0.1116 W/(m·K) for AAC400 to *λ*_0_ = 0.1888 W/(m·K) for AAC700, showing a difference of about 69%.

Increasing moisture content in each concrete led to higher thermal conductivity, which results from the progressive replacement of air in the pores by water, whose thermal conductivity (*λ* ≈ 0.58 W/(m·K)) is more than twenty times higher than that of air (*λ* ≈ 0.025 W/(m·K)).

Changing the volumetric moisture content from *U*ᵥ = 0% → 20% leads to a significant increase in the coefficient *λ* for all the tested classes. The ratios *λ(U*ᵥ = 20%)/*λ(U*ᵥ = 0%) for classes 400, 500, 600, and 700 are 3.10, 3.04, 2.79, and 2.58, respectively. It can be seen that the negative effect is more pronounced for lighter concretes with higher porosity, yet in every material it results in a significant increase in *λ*, and consequently, a drastic deterioration in its thermal insulation, from ≈260% (AAC700) to ≈310% (AAC400).

An analogous analysis performed for mass moisture content *U*_m_ yields *λ(U*_m_ = 20%)/*λ(U*_m_ = 0%) values of 1.82, 1.99, 1.99, and 2.04 for classes 400, 500, 600, and 700, respectively. So, the average increase in the λ coefficient is around 200%.

In the case of significantly higher moisture content *U*_m_ = 40% for classes 400, 500, 600, 700, the ratios *λ*(*U*_m_ = 40%)/*λ*(*U*_m_ = 0%) are 2.65, 2.99, 2.98, and 3.08, respectively. In this range, the average increase in the *λ* coefficient reaches 300%.

Analysis of the second investigated parameter shows that, for all AAC classes, the volumetric heat capacity *c*ᵥ increases linearly with rising volumetric moisture content *U*ᵥ and mass moisture content *U_m_*. The relationship is clearly linear, with good agreement (R^2^ > 0.92).

The values of the volumetric heat capacity *c*_vo_ measured in the dry state for all classes differed significantly (though less than *λ*_0_), ranging from *c*ᵥ_0_ = 0.5831 MJ/(m^3^·K) for AAC400 to *c*ᵥ_0_ = 0.8660 MJ/(m^3^·K) for AAC700, so the difference between the minimum and maximum *c*_vo_ values was 49%.

Increasing moisture content in all tested concretes led to an increase in their heat capacity *c_v_*, which was associated with the displacement of air from pores and filling them with water, which has a very high volumetric heat capacity of about 4.18 [MJ(m^3^∙K)] (at room temperature).

By conducting a similar analysis as above but concerning the variability of the second parameter *c_v_* with volumetric moisture *U_v_*, we get the following relationships for the classes 400, 500, 600, and 700: *c_v_*(*U_v_* = 20%)/*c_v_*(*U_v_* = 0%) = 2.76, 2.59, 2.04, and 1.67. So, the increase in the *c_v_* parameter with *U_v_* in this range is from about 170% (AAC700) to around 280% (AAC400).

By making a similar assessment for mass moisture content *U_m_*, we obtain the following values for the different classes 400, 500, 600, and 700: *c_v_*(*U_m_* = 20%)/*c_v_*(*U_m_* = 0%) = 1.70, 1.77, 1.58, and 1.43. So, the increase in the *cᵥ* parameter caused by the change *U_m_* = 0% → 20% ranges from about 140% to about 180%.

For substantially higher moisture content (*U_m_* = 40%), the ratios *cᵥ(U_m_* = 40%)/*cᵥ(U_m_* = 0%) are 2.41, 2.54, 2.16, and 1.87 for classes 400, 500, 600, and 700, respectively. The increase in *cᵥ* within the range *U_m_* = 0% → 40% is roughly between ≈ 190% and ≈ 250%.

The data presented above show that even a moderate increase in moisture content leads to a significant rise in both the thermal conductivity *λ* and the volumetric heat capacity *c_v_* for each of the tested concretes (AAC400, AAC500, AAC600, AAC700). Therefore, it is absolutely necessary to take the level of moisture into account when performing calculations related to both the thermal insulation and the thermal dynamics of building partitions made with autoclaved aerated concrete.

The studies conducted showed very good accuracy of the linear description for the function *λ* = *f*(*U_m_*) and for the function *λ* = *f*(*U_v_*). Therefore, the linear function is recommended for the studied group of materials (AAC400, AAC500, AAC600, AAC700) when describing the relationship of their thermal conductivity with both mass moisture content *U_m_* and volumetric moisture content *U_v_*. Similar conclusions apply to the dependence of heat capacity on moisture content. The results also confirm, in the case of this second thermal parameter, the possibility of using a linear function to describe its relationship with mass and volumetric moisture content. It has been shown that for all tested classes, good accuracy is achieved both for the function *c_v_* = *f*(*U_m_*) and *c_v_* = *f*(*U_v_*). Therefore, for the studied group of materials (AAC400, AAC500, AAC600, AAC700), the linear function is recommended for describing the relationship of heat capacity *c_v_* with mass moisture content *U_m_* and volumetric moisture content *U_v_*.

In the next stage of research, it is planned to connect the results presented in this article with the findings published in previous studies [20,21]. In study [20], the sorption isotherms of four tested aerated concretes were determined using the DVS technique and the traditional method. Then, the applicability of various models to describe sorption isotherms of this material group was been extensively evaluated in work [21]. The tested models (Peleg, Redlich, Chen, Oswin, Henderson, Lewicki, Caurie, and GAB) all provided a very good fit with the experimental results for the tested material group. The authors are currently working on reproducing the variability of both thermal parameters (*λ* and *c_v_*) of the tested classes in relation to their sorption isotherms. This will make it possible to use the results presented in this article for any operating humidity.

## Figures and Tables

**Table 1:** Parameters of the tested autoclaved aerated concretes declared by the manufacturer, prepared based on factory labels.

Density Class	400 [kg/m^3^]	500 [kg/m^3^]	600 [kg/m^3^]	700 [kg/m^3^]
Compressive strength [N/mm^2^]	≥2.0	≥2.5	≥3.0	≥4.0
Strength class	2.0	2.5	3.0	4.0
Thermal conductivity λ_10_ [W/(m·K)]	0.110	0.140	0.170	0.200

## Data Availability

The original contributions presented in the study are included in the article, further inquiries can be directed to the corresponding author.
